# Key nodes affecting patient satisfaction in a cross-regional referral service process: an empirical analysis study in Sichuan

**DOI:** 10.1186/s12913-018-3460-8

**Published:** 2018-11-07

**Authors:** Xinli Zhang, Tianjin Wang, Yu Yu, Shuzhen Zhao

**Affiliations:** 1 Business School of Sichuan University, Chengdu, 610065 China; 2 West China Hospital of Sichuan University, Chengdu, 610041 China

**Keywords:** Referral service, Satisfaction, Logistic regression, Peak-end rule

## Abstract

**Background:**

The referral service is a significant component of healthcare reform in China, and the measurement of patient satisfaction with the referral service process will help to improve the quality of referral medical delivery. Furthermore, the referral service in China includes inter-institutional collaborations between hospitals at different levels and multi-nodes throughout the referral process. It is therefore necessary to identify the key nodes that affect patient satisfaction during the referral service process.

**Methods:**

This study conducted a questionnaire survey of 110 patients to collect data regarding patient satisfaction at the following healthcare nodes: primary-level hospital, referral appointment registration, claim of appointment number in the outpatient department, examination service, admission service, and overall satisfaction during the referral service process. Correlation analysis and logistic regression methods were used to establish a mathematical model of patient satisfaction between five nodes and overall satisfaction. Additionally, a peak-end model was formed to identify the peak node impacting overall patient satisfaction during the referral service based on the sample data.

**Results:**

Over 80% of referral patients rated the overall referral service as ‘good’. The correlation analysis revealed that there was a significant correlation between the satisfaction of each node and the overall satisfaction (*P* < 0.05). The results of the regression model showed that the satisfaction of five nodes determined the overall satisfaction and that “admission service at the higher-level hospital” exerted the greatest impact on overall satisfaction (β = 0.312), while “referral appointment registration” had the lowest influence on overall satisfaction (*β* = 0.177). The peak-end model also revealed that “admission service at the higher-level hospital” had a greater effect on overall satisfaction.

**Conclusion:**

Our study showed that the key nodes affecting patient satisfaction were “transferring service at the primary-level hospital” and “admission service at the higher-level hospital”. Furthermore, the efficacy of the referral services is determined by the gatekeepers’ management of the referral system at the primary-level hospital and the allocation and management of bed resources at the higher-level hospital. These findings can serve as a science-based guidance for them to improve their performance in inter-regional healthcare collaborations in the referral service process.

## Background

Referral service is a patient-transferring process that is conducted between hospitals at different levels, such as specialty hospitals and comprehensive hospitals, or among specialty hospitals [1]. The referral process in this study indicates patients with serious illness being transferred from primary-level hospitals to higher-level hospitals, which is a key process to share healthcare resources between different regions and is the core of the hierarchical medical system, addressing issues such as the provision of difficult medical services and expensive medical costs [2]. In China’s medical reform process, improving referrals is a priority in the improvement of healthcare services, and efficiency of the referral process is also a focus of research in healthcare service management. Furthermore, the Chinese government has recognized the importance of the referral service and has made great efforts to establish and optimize the referral flow [3]. However, the effect of these efforts has been unsatisfactory.

Some scholars have explored which factors have an effect on the referral process. Li Zhenyu [4] concludes that a dysfunctional operation model of hospitals and the limited awareness of healthcare personnel and patients are the main factors. Furthermore, the satisfaction of patients has become a very important index to evaluate hospital management quality and medical quality [5], which has led to additional research focusing on the satisfaction of patients during the referral process, aiming to establish a well-ordered referral service system. Tejal K. Gandhi found that the principal factor affecting treatment and lowering patient satisfaction during referral is the delay and inaccuracy of information [6]. Denys Greenhow has shown that patients have a desire to participate in referral decisions and are willing to be informed about related information, which could increase patient satisfaction [7]. Jonathan S Einbinder demonstrated that understanding patient preferences for experts can improve the efficacy of the referral process [8]. In addition, the establishment of platforms for referral information and recommendations by experts have a positive influence on the improvement of the referral process and patient satisfaction. Mogere Dominic M analysed the referral process and found that the timeliness and integrity of information delivery and the choice of hospital were the primary factors influencing patient satisfaction [9].

The studies above indicate that the research approach of studying the inter-regional referral service has shifted from simply finding factors that impact overall satisfaction of the referral service to the process management of the referral service; however, there have been limited studies regarding patient satisfaction at different nodes during the referral process. In China, the delivery of healthcare services involves five independent referral nodes, namely, the transferring service at the primary-level hospital, referral appointment registration, claim of appointment number in the outpatient department, examination service, and admission service. Therefore, this study aimed at finding correlations between the overall satisfaction of patients during the referral service and patient satisfaction at each node of the referral process.

According to the rational action theory, there is a correlation between the overall satisfaction of patients throughout the referral service and their satisfaction at each node of the referral process [10]. He Da’an argues that there is an analysis chain during rational decision-making in real life that extends from the cognitive process to the adjustment of utility expectation [11], and this has inspired researchers to establish links between the cognitive process and utility expectation. In other words, based on a new interpretation of the rational action theory, we can analyse the relationship between the cognitive process and utility expectations due to the different referral nodes where patients’ emotions and feelings will change along with experiencing various services at the different nodes during the referral service process, which can lead to the adjustment of patients’ overall utility evaluation after experiencing the entire referral process.

Considering that satisfaction is generally selected to evaluate patients’ emotions and feelings, we can explain the mechanism by which the cognitive process and experience affects the overall utility evaluation through this framework. Therefore, we postulate the following assumptions: (1) The evaluation of service at referral nodes affects overall satisfaction; and (2) the influence that different nodes have on the overall satisfaction of the referral process varies, and we will try to verify this using an empirical study. The main aim of the current study is to assess the utility of the nodes on the overall satisfaction of the referral service and to find the key nodes.

**Fig. 1:**
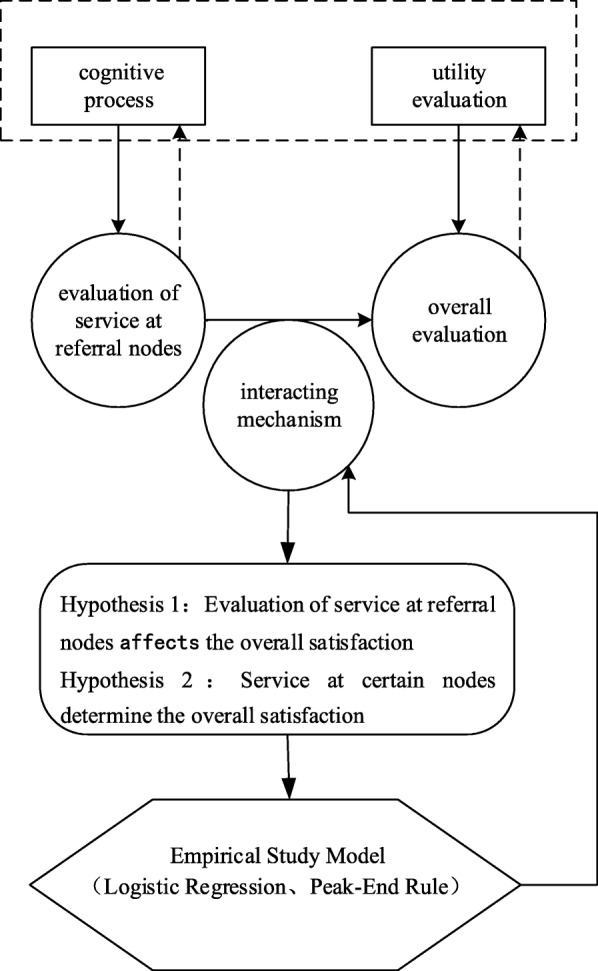
Research flowchart

The research hypothesis testing and empirical analysis flowchart is shown in Fig. 1.

**Fig. 2:**
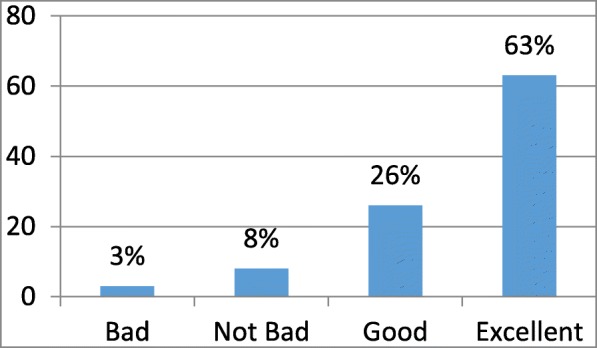
Distribution of overall satisfaction with the referral service

## Methods

### Study setting and design

This study was conducted at the West China Hospital of Sichuan University Hospital of Chengdu, China, from June to July 2017. The West China Hospital is one of the first hospitals in China to implement a referral policy and has signed contracts with 31 community hospitals as of January 23, 2015 [12]. Our study population consisted of patients who experienced all nodes of the referral process. The study sampled patients at random to conduct a questionnaire survey, the content of which was mainly focused on the referral service process in China, including the patients’ overall satisfaction with the referral service process, the transferring service at the primary-level hospital, the referral appointment registration, the claim of appointment number in the outpatient department, the examination and the admission process.

### Sample size

Sample size was calculated by using a single population proportion formula. It was calculated using a 95% confidence interval, a marginal error of 8%, and a patient satisfaction of 80%. There was an added contingency of 10% for non-response and inappropriate response, and this yielded a sample size of 110. The systematic random sampling technique was used to select patients who experienced the entire referral process during the study period until the predetermined sample size was reached.

### Data collection tool and procedures

The subjects in the study included patients who experienced the entire referral process during the study period. Written informed consent was obtained from the patients who were willing to participate in the study, and a questionnaire was conducted with the patients. Data regarding patient satisfaction at different nodes and their overall satisfaction of service throughout the referral service process in West China Hospital was collected using a structured standardized questionnaire.

The questionnaire included the patients’ overall satisfaction with the referral service process, satisfaction with the transferring service in the primary-level hospital, satisfaction with referral appointment registration, satisfaction with claim of appointment number in the outpatient department, satisfaction with the examination and satisfaction with admission. On the questionnaire scale, “1” rated the item as “bad”, while “2”, “3” and “4” indicated “not bad”, “good” and “excellent”, respectively.

### Analysis

The collected data were exported into SPSS (Windows version 21) for analysis. As shown in Fig. 2, over 80% of the referral patients rated the overall referral service as good. In terms of overall satisfaction, the study utilized the mean, median and mode of the sample data to reflect the concentration trend of satisfaction of each evaluation indicator. The minimum mean, minimum median and mode of the various indicators was 88, 93, and 92.5, respectively, which showed that the patients were satisfied with the current referral process. To reflect the satisfaction trends of each evaluation indicator, the study used the standard deviation, partial kurtosis and skewness of the samples of the minimum mean, minimum degree of data dispersion and degree of deviation, respectively. Based on the results in Table 1, it can be concluded that all of the satisfaction indicators fluctuate primarily in the range of 80–100.

**Table 1:** Statistical analysis of patient satisfaction at different nodes

Statistics		satisfaction with the transferring service in the primary-level hospital (hundred-mark system)	satisfaction with referral appointment registration (hundred-mark system)	satisfaction with claim of appointment number in the outpatient department (hundred-mark system)	satisfaction with the examination (hundred-mark system)	satisfaction with admission (centigrade)	Overall satisfaction with the referral process
N	Valid	100	100	100	100	100	100
	Missing	4	4	4	4	4	4
Mean		91.020	88.930	90.780	90.660	90.200	3.490
Median		95.000	92.500	95.000	95.000	90.000	4.000
Mode		100.0	100.0	100.0	100.0	100.0	4.0
Standard deviation		11.079	13.213	12.188	13.805	11.131	.772
Skewness		−1.466	−1.230	−1.640	−3.470	− 1.181	− 1.512
Standard error of skewness		.241	.241	.241	.241	.241	.241
Kurtosis		2.137	.760	2.283	18.563	.767	1.759
Standard error of kurtosis		.478	.478	.478	.478	.478	.478

An empirical analysis model was established based on correlation analysis and logistic regression methods to identify the utility of the referral nodes to the overall satisfaction of the referral process, which enabled us to identify the key nodes. In addition, the peak-end rule was used to find the peak node of the service process. Finally, the key nodes that affected the overall satisfaction in the referral service process were identified by comprehensively comparing the results of the two methods.

### Logistic regression method modelling process

To verify Hypothesis 1 and Hypothesis 2, a logistic regression model (shown below) was constructed to test the relationship between satisfaction with the service at referral nodes and overall satisfaction.

1 $$ Y=\sum \limits_{j=1}^5{\beta}_0+{\alpha}_j{X}_j+{\varphi}_j\left(j=1,\mathrm{2.3...5}\right) $$

where *j* is the number of the referral service node, *β*_*0*_ represents a constant term, *α*_*j*_ indicates the regression coefficient, and φ_j_ represents the random error of each variable. The variable *X* and Y are explained in detail in Table 2, below.

**Table 2:** Variable assignment

Process evaluation element	Variable Name	Variable assignment
Overall satisfaction	Y	1 = bad,2 = not bad,3 = good,4 = excellent
Satisfaction with transferring service at the primary-level hospital	X_1_	hundred-mark system
Satisfaction with referral appointment registration	X_2_	
Satisfaction with claim of appointment number	X_3_	
Satisfaction with examination	X_4_	
Satisfaction with admission	X_5_	

### Peak-end rule method modelling process

An influential study by Redelmeier and Kahneman (1996) demonstrated that patients’ memories of the amount of discomfort reported after an acute minimally invasive procedure was determined primarily by the intensity of pain at both the procedure’s worst and most recent episodes, a phenomenon now known as the “peak-end rule”. Experiences and memories are often mismatched [13]. The theory holds that people’s memory of an experience is determined by the peak and the end of the experience. The peak refers to the maximum value and the minimum value, and the end refers to the value at the end. Recently, the theory has been often used to evaluate the satisfaction of service, and it has been able to find the influencing factors in order to improve patient experience [14]. According to this theory, the peak and end values determine a patient’s satisfaction with the healthcare service provided. That is to say, if the patient felt satisfied with the peak and final experiences of the healthcare service, they were more likely to feel satisfied with the entire service process; therefore, the most extreme (peak) and final (end) impressions are often better predictors of overall evaluations of experience than average impressions [15]. According to Kahneman, the overall satisfaction value of a single sample, b_i_^′^, can be determined by Eq. (2) [16].

2 $$ {b_i}^{\prime }=\frac{\max\ {b}_{ij}+{b}_{is}}{2} $$

where the referral nodes are indicated by j∈ (1,s-1). In this case, s = 5.

The variable max b_ij_ represents the peak, i.e., the maximum value of satisfaction of the i-th patient after he or she experiences (s-1) nodes. The variable b_is_ is the final value, representing the value of satisfaction at the final node of the referral process.

## Results

### Correlation relationship between the overall satisfaction and the satisfaction of service at each referral service node

The results of the correlation test between the patients’ overall satisfaction and their satisfaction at each node along the referral service process is shown in Table 3.

**Table 3:** Correlation between overall satisfaction and the satisfaction at each node

Referral service node	Correlation	Significance (two-tailed)	df
Evaluation of transferring service at the primary-level hospital	0.449	0.000	94
Referral appointment registration	0.208	0.042	94
Claim of appointment number in the outpatient department	0.276	0.006	94
Medical examination	0.314	0.015	94
Admission at the higher-level hospital	0.597	0.000	94

Table 3 shows *p* < 0.05 in the all correlation significance tests, which indicates that all of the nodes were statistically significantly correlated to overall satisfaction. In addition, the coefficient between admission at the higher-level hospital and overall satisfaction was the largest at 0.597. The minimum coefficient of correlation was 0.208. This comparison demonstrated that hypothesis (1) is established, that is, the satisfaction at each referral service node affects overall satisfaction. Furthermore, the degree to which each node could affect overall satisfaction varied.

### Regression relationship between overall satisfaction and the satisfaction with service at each referral service node

Table 4 shows that the model’s coefficient of determination (R^2^) was 0.845, and the adjusted R^2^ = 0.836 when considering the degrees of freedom. In other words, the predicted value of the dependent variable explained 84.5% to 95% of the information in the model, which meant the model fitting based on the sample data was well explained. The F-test variance analysis with 95% confidence intervals produced *p* < 0.05, which indicated that the model was reliable.

**Table 4:** The coefficients of the regression model of satisfaction with service at referral nodes and overall satisfaction

Constant variable	Non-standardized coefficient		Standardized coefficient	t-value	Sig.	95.0% confidence interval of β	
	β	Standard error	Trial edition			Lower limit	Upper limit
(constant)	−3.2	0.302		−10.67	0	−3.817	− 2.619
Referral to higher-level hospital	0.02	0.004	0.276	4.878	0	0.011	0.027
Referral appointment registration	0.01	0.003	0.109	2.059	0.042	0	0.012
Claim of appointment number at the outpatient department	0.01	0.004	0.182	2.784	0.006	0.003	0.02
Medical examination	0.01	0.003	0.167	3.202	0.002	0.004	0.015
Admission process	0.03	0.004	0.4	7.215	0	0.02	0.035

Based on Table 4, we constructed the model in eq. (3),

3 $$ Y=-3.218+0.019{X}_1+0.006{X}_2+0.012{X}_3+0.009{X}_4+0.028{X}_5 $$

In the multi-collinearity test of the model, the significance probabilities of the t-values were, respectively, 0.000, 0.042, 0.006, 0.002, and 0.000 when the explanatory variables in the five regression calculations were related to the satisfaction at the referral service nodes (transferring service at the primary-level hospital, referral appointment registration, claim of appointment number, admission process, and medical examination). Additionally, the constant t-value was 0.000. Therefore, the t-value of all the variables was less than 5%, which showed that the corresponding coefficient had a significant difference from 0. The results of Spearman’s correlation coefficient test are shown in Table 5.

**Table 5:** Spearman’s correlation coefficient test results

|e|			
Spearman rho	Satisfaction with the referral to higher-level hospital	Correlation coefficient	.312
		Sig. (two-tailed)	0.06
		N	100
	Satisfaction with the referral appointment registration	Correlation coefficient	0.177
		Sig. (two-tailed)	0.077
		N	100
	Satisfaction with the claim of appointment number in the outpatient department	Correlation coefficient	.304
		Sig. (two-tailed)	0.088
		N	100
	Satisfaction with medical examination	Correlation coefficient	.257
		Sig. (two-tailed)	0.09
		N	100
	Satisfaction with admission	Correlation coefficient	.197
		Sig.(two-tailed)	0.05
		N	100

The table above shows that Spearman’s correlation coefficient between|e|and satisfaction with the transferring service at the primary-level hospital, satisfaction with referral appointment registration, satisfaction with claim of appointment number in the outpatient department, satisfaction with the examination and satisfaction with admission was 0.312, 0.177, 0.304, 0.257, and 0.197, respectively. There were significant differences between the nodes, signifying non-existence of heteroscedasticity.

The regression model showed that the evaluation of the admission service, transferring service at the primary-level hospital, claim of appointment number, medical examination service and referral appointment registration service determined the evaluation of the overall referral service. The importance of the different nodes in the evaluation of the overall referral process is listed below according to the linear correlation coefficients.

4 $$ {\upbeta}_5>{\upbeta}_1>{\upbeta}_3>{\upbeta}_4>{\upbeta}_2 $$

where β_5_, β_1_, β_3_, β_4_and β_2_, respectively, represent the evaluation of the admission process at the hospital, the transferring service at the primary-level hospital, the claim of appointment number, the medical examination at the hospital and the referral appointment registration.

Formula (4) is a proof of hypotheses 2. In the current referral service, the admission at the higher-level hospital (i.e., the end of the referral process) exerted the greatest impact on overall satisfaction, while the referral appointment registration had the least influence on overall satisfaction.

### Empirical study of the peak-end model

Based on formula (2), the total satisfaction of samples, *b*_*i*_^′^, in the peak-end rule was calculated. The average satisfaction of 100 patients at four nodes was calculated, as denoted by *b*_*j*_(j = 1, 2, 3, 4), and was 91.22, 89.56, 91.07, and 90.84, respectively. The value *b*_1_, the transferring service at the primary-level hospital, was the largest value, with a frequency of 75%. Therefore, we designated node 1 as our peak node.

**Fig. 3:**
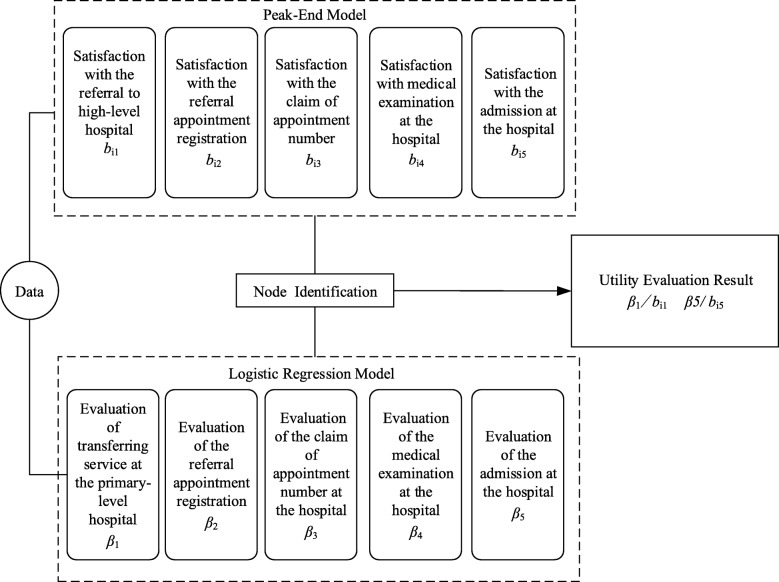
Empirical research framework of patient satisfaction with the referral process

On the other hand, the significance p for the total peak-end rule as determined by the formula was less than 0.05. According to Table 1, the correlation coefficient of the final node was 0.597, and the correlation coefficient of the peak node was 0.449, which were both higher than the rest of the three nodes. The correlation coefficients and *p*-values indicated that patient satisfaction adhered to the peak-end rule during the referral process. The final empirical research finding of the two models employed in our study is shown in Fig. 3.

In this study, it was proven by the correlation test that patient satisfaction with each node of the referral process was significantly related to overall patient satisfaction. The regression model further analysed the relationship between satisfaction with the nodes during the referral process, and the results showed that satisfaction with admission, satisfaction with the referral service at the primary-level hospital, satisfaction with claim of appointment number, satisfaction with the medical examination service and satisfaction with referral appointment registration all had a significant effect on overall satisfaction. By employing the peak-end rule to analyse the satisfaction with the referral service nodes, we found that the final node (admission at the higher-level hospital) had the greatest impact on overall satisfaction, followed by the peak node (transferring service to the primary-level hospital).

## Discussion

This study showed that both the regression model and the peak-end model produced the same results, which indicates that the peak-end rule, generally used in the service process, was also suitable for studying the referral service process. There have been a substantial number of studies regarding the factors that impact patients’ overall satisfaction but relatively few regarding the key nodes of patient satisfaction during the referral service process. Therefore, this study gives new insights into the field of referral process management.

The research framework had two valuable aspects of practical significance. One aspect was the different influences that the referral service nodes had on overall satisfaction because the phenomenon that patient satisfaction varies with the different referral service nodes could be explained by the rational behavioural decision theory, as previously mentioned. That is, the patients obtained information throughout the rational decision-making process, and simultaneously, their experiences at the different referral nodes had a heuristic effect on them, which resulted in different experience deviations. The utility evaluation of overall service was therefore adjusted to a utility evaluation of particular service nodes, and the experiences impacting the patients’ cognitive process determined their final judgement. The referral nodes included the transferring service at the primary-level hospital, referral appointment registration, claim of appointment number at the outpatient department, medical examination at the hospital, and admission at the hospital, where the transferring service at the primary-level hospital and the available beds at the higher-level hospital were key nodes. The findings also followed the peak-end rule. In addition, the results of another study have shown that if measures were adopted to manage available beds at the higher-level hospital, patient satisfaction could be further improved. Similarly, the decision-making efficiency of the gatekeeper at the primary-level hospital was an important indicator that had an enormous influence on the experience of patients [17].

Another aspect of practical significance of this research was that the overall evaluation of the referral process was influenced by the actions of both the primary-level hospital and the higher-level hospital, which has also been approved by the experts of referral department of West China Hospital of Sichuan University. Moreover, according to the rational decision-making theory, patients made their decisions by taking the information and environment into consideration based on the promotion of their experience utility. That is to say, patients’ decision would alter in response to their satisfaction with the collaboration between the different hospitals. Therefore, medical service organizations of different levels should establish collaborative standards in terms of system, environment and service so as to enhance the experience of patients. Similarly, research by Karemere H [18] also demonstrated that referral performance depends on the institutional arrangements of the intermediary agency and their ability to adapt to demands. Additionally, Kurtzman [19] focused on the impact of the regional environment on the practice of healthcare practitioners and found that the referral environment is a factor influencing the service provided by nurses; therefore, local referral services should be conducted in an organized manner.

This study had certain limitations. First, we did not consider individual differences in the statistical model and calculations. For example, the satisfaction research on subjects with demographic differences carried out by Surur AS et al. [20] is a follow-up to the difference study of the satisfaction of patients based on satisfaction with the referral process. Although the representation of the sample was guaranteed by factors such as the gender balance, health balance, and source level balance of patients, the patients’ personal preference would eventually affect the satisfaction results. Second, in this paper, we only used the method of calculating the average degree of satisfaction to obtain the peak value and did not analyse the sample’s satisfaction deviation at the peak node. Therefore, the calculation method of the peak value in the peak-end model will be a keystone of a future study.

## Conclusion

The results of both the traditional regression method and the new peak-end rule method show that the overall satisfaction with the referral service is affected by the division of labour in key nodes of inter-institutional service. The key nodes affecting patient satisfaction were “transferring service at the primary-level hospital” and “admission service at the higher-level hospital”. This highlights the need to strengthen services in these areas. In summary, our research has implications in the following two aspects. For the service process, the improvement of patient satisfaction during the referral service is determined by the gatekeepers’ management of the referral system at the primary-level hospital and the allocation and management of bed resources at the higher-level hospital, which is the result of joint efforts by hospitals at the different levels. Regarding the referral system, the findings can provide recommendations for practical medical reforms. First, hospitals at the different levels should clarify roles and perform their individual functions to foster cooperation and ensure the efficient execution of the referral service process. Second, hospitals should focus on the improvement of service at the nodes of referral appointment registration, claim of appointment number in the outpatient department, and examination service to promote patient satisfaction in details.
